# Turkish teachers’ values with rational and non-rational truth and teacher emotions in teaching

**DOI:** 10.3389/fpsyg.2024.1395920

**Published:** 2024-12-04

**Authors:** Kübra Ünal, Liisa Myyry, Auli Toom

**Affiliations:** ^1^Faculty of Social Sciences, University of Helsinki, Helsinki, Finland; ^2^Department of Education, Faculty of Educational Sciences, University of Helsinki, Helsinki, Finland; ^3^Centre for University Teaching and Learning, Faculty of Educational Sciences, University of Helsinki, Helsinki, Finland

**Keywords:** personal values, teachers, emotions, Schwartz’s value model, truth-related values, gender differences

## Abstract

The aim of this study was to clarify the relationships between personal values, truth-related values and emotions among Turkish teachers. The Schwartz’s Value Theory and Frenzel’s teacher emotion model were used as the theoretical framework. This study used a cross-sectional correlational research design. The data were collected from 279 teachers with the Portrait Value Questionnaire (PVQ) where rational truth and non-rational truth values were added, and with the Teacher Emotions Scale (TES). The circular structure of the Schwartz Value Theory was tested by multidimensional scaling. The data analysis aimed to uncover relationships between personal values, truth-related values, and emotions. Rational truth emerged near self-direction and self-transcendence, yet items measuring non-rational truth were scattered among values. Females had higher regard for self-direction and hedonism than males. Non-rational truth was negatively correlated with enjoyment, while it was positively correlated with anxiety. The implications for gender roles in Turkish society are discussed.

## Introduction

Many social scientists perceive that values have a fundamental importance in explaining human emotions and behaviors (Schwartz, 1992; Kusdil and Kagitcibasi, 2000; Schwartz and Bardi, 2001). Values have been the subject of numerous research (Cieciuch and Davidov, 2012; Demirutku and Sumer, 2010; Roccas and Sagiv, 2017; Schwartz and Butenko, 2014; Schwartz and Cieciuch, 2022) as a guiding concept in explaining cognitive and social structures, processes, and social behaviors in different disciplines. Values are also found to be related to professional behavior (Knafo and Sagiv, 2004) and guiding teachers’ decision-making and their justification (Pajares, 1992). Teachers are important socialization agents in their cultures (Schwartz, 1992; Tamm et al., 2020) and role models to children (Thornberg and Oguz, 2016), and thus, their values are essential to examine. The research on teacher education has recently highlighted the importance of teacher-related individual characteristics including motivation, job satisfaction, self-efficacy, and emotions which increase the effectiveness of teaching and learning at schools (Alpaslan and Ulubey, 2017). Within the framework of professional competence in teaching, emotions have a very important role as they are highly influenced and shaped by surrounding the environment while shaping the professional actions and decisions as well as the personal growth of a person (Aral and Mede, 2018). In addition, teachers’ emotions affect their instructional behaviors (Becker et al., 2014). There is a wide spectrum of value issues, scrutinizing the motivational goal of values differentially and developing the value list with added (truth-related) values, which form a cross-culturally stable motivational continuum (Ahola, 2017), but they have been the subject of very scarce studies. In this study, teacher values are examined via the lens of truth-related values and explained with their emotions, which is the first empirical attempt to investigate this relationship among teachers.

Values are known to influence the country’s political structure, education system, workplace productivity, and social welfare (Tatto, 2019). One explanation for cultural variation could be cultural tightness or looseness. This dimension, developed by Gelfand et al. (2011) and Uz (2015), refers to the normative pressures in a culture. While social norms are weak and deviant behavior is tolerated more in loose cultures, tight cultures have numerous strong norms and less tolerance for them. Considering the education and teachers in Türkiye^1^, respect for spiritual values has come into force to be gained by students in all kinds of educational activities in the Turkish Basic Law of National Education since 1973 [Ministry of National Education (MoNE), 1973]. Hence, Türkiye, where cultural homogeneity is at a high level, represents a tight culture (Gelfand et al., 2011; Uz, 2015). The aim of this study is to examine, how the two motivational constructs, personal values and emotions, are related to each other among Turkish teachers.

### Personal values

Currently, Shalom Schwartz’s (1992) theory of universal content and structure of values is the most widely used in value research. According to the theory, values are goals and motivations, which serve as the guiding principles of people’s lives. As cognitive representations of abstract goals, values motivate people to attain different interests. Universality in the theory means that the meaning of values (content) and their location in the value model (structure) are approximately the same in different cultures. Besides the cognitive component, values include an affective component, and these two components are interconnected. Thus, desiring the goal (e.g., success) indicates that one has a positive effect toward it (Schwartz, 1992).

Schwartz’s theory defines values as organized into 10 universal types that serve different interests or motivational goals. Values and their contents are presented in Table 1.

**Table 1:** Schwartz’s basic values and their contents (Schwartz, 1992).

Value	Content
Power	Societal prestige and controlling others
Achievement	Personal success and competence according to social norms
Hedonism	Pleasure and the satisfaction of sensual needs
Stimulation	Excitement, novelty and challenge in life
Self-direction	Independent action and thought, making one’s own choices
Universalism	Understanding, tolerance and protection for the welfare of all people and for nature
Benevolence	Protecting the welfare of close others in everyday interaction
Tradition	Respect, commitment and acceptance of the customs and ideas that one’s culture or religion impose on the individual
Conformity	Restraint of actions, inclinations and impulses likely to upset or harm others, or violate social expectations or norms
Security	Safety, harmony and the stability of society, of relationships and of self

**Figure 1:**
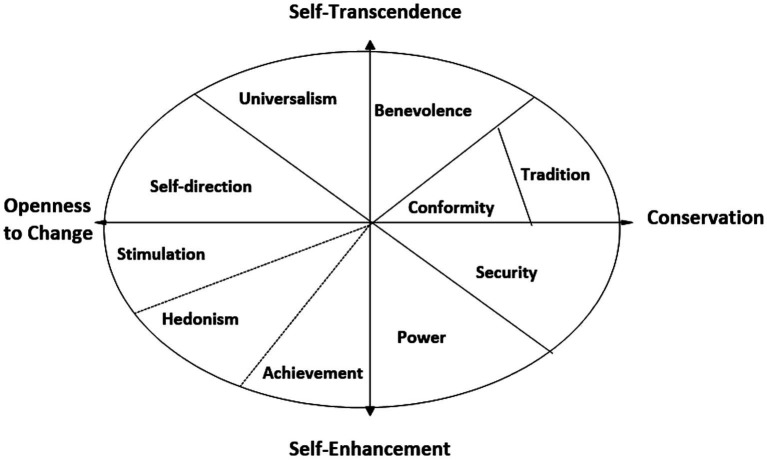
Schwartz’s model of motivational types of values. Reproduced with kind permission from Elsevier © 1992.

According to Schwartz’s (1992) theory, the goals and interests that values serve can be either compatible or conflicting with each other, and based on these compatibilities and conflicts they form a special two-dimensional circular structure on two levels. Firstly, values can serve either individual or collective interests. Power, achievement, hedonism, stimulation, and self-direction are values that serve individual interests; and benevolence, tradition, and conformity serve collective interests. Universalism and security serve both interests and are situated in the boundaries between these two. Secondly, values structure forms two main dimensions, self-transcendence vs. self-enhancement and openness to change vs. conservation (Figure 1). The former represents the extent to which people are motivated to transcend selfish concerns and promote the welfare of others (including such values as benevolence and universalism) compared to enhancing their own personal interests even at the expense of others (power and achievement values). The latter relates to the motivation to follow one’s own intellectual and emotional interests (self-direction, stimulation, and hedonism values), compared to preferring the status quo and the certainty provided by relationships with close others, institutions, and traditions (tradition, conformity, and security values). Correlations between values and other variables should demonstrate a sinusoid pattern: for example, empathy shows the highest (positive) correlation with universalism and the lowest (negative) with power, and the remaining correlations increase and decrease systematically as one moves along the circle (Myyrya et al., 2010). The multidimensional structure of values has reached empirical support in different cultures (e.g., Schwartz and Boehnke, 2004).

In Schwartz’s (1992) original study and presentation of the value theory, the main target group was schoolteachers because they, according to Schwartz (p. 18), “play an explicit role in value socialization, they are presumably key carriers of culture, and they are probably close to the broad value consensus in societies.” Recent studies in different cultures show that self-transcendence and openness to changes values seem to be high in teachers’ value hierarchy whereas self-enhancement and conservation values are lower (Barni et al., 2019; Marušić-Jablanović, 2018; Perrin et al., 2021; Tamm et al., 2020). Previous studies in the Turkish context confirm the global empirical data (Ceylan and Yildiz, 2019; Uzun, 2018); however, recent studies show that power, achievement and tradition (Tekin, 2021) and hedonism (Gonluacik et al., 2022) are most preferred value types among Turkish teachers.

### Truth related values

Schwartz’s (1992) model presents values that are found to be universal by both the content and structure. Less is known about the values that are motivationally mixed and/or non-universal. Wach and Hammer (2003) proposed two truth-related values to be added to Schwartz’s value survey: rational truth and non-rational truth. Rational truth refers to theoretical, logical, and predictable truths and non-rational truth refers to belief in magic, intuitiveness, fatalism, and the denial of rationality (Ahola, 2017), pietism (Emre and Yapici, 2015; Yapici et al., 2012) and hospitality, secularism, and male privilege (Kusdil and Kagitcibasi, 2000). The sparse research concerning truth-related values shows that rational truth is located near universalism and self-direction in the Schwartz model whereas non-rational truth has been close to security, tradition, and power (Ahola, 2017; Wach and Hammer, 2003). Ahola (2017) concludes that more studies about truth-related values are needed, and they are especially worth considering when investigating knowledge-related topics. Because teachers possess an important role in educating new generations, it is crucial to examine how truth-related values are related to other values and how they are regarded in teacher samples.

### Gender differences in values

It has been discovered that, for both genders, the arrangement of single values within value types and the structure of value types are strikingly similar across a wide range of civilizations (Prince-Gibson and Schwartz, 1998; Struch et al., 2002). Concerning the value preferences, females have generally valued benevolence and universalism more than males, and males have usually higher regard for power and achievement than females (e.g., Papastylianou and Lampridis, 2016; Smith and Schwartz, 1997). Results obtained in the Turkish context are mixed: in some studies, gender does not affect value differences (Imamoglu and Karakitapoglu-Aygun, 1999), whereas some reveal that gender is an important determinant of value differences (Demirutku and Sumer, 2010; Firat and Acikgöz, 2012; Kusdil and Kagitcibasi, 2000; Yilmaz, 2009).

All these results may be interpreted to mean that the Turkish schoolteachers’ values in meaning are identical across genders; however, it seems that among them the gender differences might be a bit different from those observed in other cultures. Yilmaz (2009) found that female teachers had higher regard for self-transcendence and conservation values than males. In Aktay and Ekşi, 2009 study female teachers preferred to score on self-direction more than males whereas males scored on universalism more than females. In addition, there exists research in the Turkish context that females gave more importance to hedonism and security than males (Firat, 2008; Memis and Gedik, 2010).

### Teachers’ emotions

A recently applied model to study teacher emotions is Frenzel (2014) and Frenzel et al. (2016) Teacher Emotions Scale (hereafter TES). According to appraisal theory (e.g., Moors et al., 2013), it is based on the idea that emotions are primarily caused by individuals’ subjective cognitive judgments about significant situations and events rather than by the situations and events themselves (Frenzel, 2014). The model considers the three main emotions in teaching to be enjoyment, anger, and anxiety, which are measured by the TES. The TES sees emotions as states more than traits, i.e., temporary experiences, instead of more stable and general feelings (Frenzel et al., 2016). Pleasure derived from either a previous (outcome-related joy) or an upcoming (anticipatory joy) occurrence, or from indulging in a pleasurable activity, is referred to as enjoyment. For enjoyment, the main source seems to be students’ success (Frenzel, 2014). Anger is claimed to be the most prominent negative emotion in teaching (e.g., Sutton and Wheatley, 2003), although it might be reported less than it is felt because it is not socially acceptable (Frenzel, 2014). Anger can be caused by blaming others for unwanted incidents (Smith and Lazarus, 1993), for instance, disobedient pupils, or being discontented with one’s own behavior (Frenzel, 2014). Threat and incapability to cope with it, evokes anxiety (Smith and Lazarus, 1993). Anxiety can be felt due to poor preparation for teaching or problems with discipline in the classroom, and it is found to be more typical among young teachers (Frenzel, 2014).

Teacher emotions are closely associated with not only teachers’ well-being (Gross and John, 2003) but also the quality of education at schools (Alpaslan and Ulubey, 2017; Sutton and Harper, 2009). Likewise, the teachers’ academic emotions, in which love (Hargreaves, 2005) and enjoyment (Chen, 2016) are mentioned as the most frequent emotional labor by teachers in class, are very significant for achieving the goals and mission of education (Shariatmadari et al., 2019).

### Relationships between personal values and emotions

Because personal values have both cognitive and affective components, they trigger feelings when they are activated; for instance, high regard for independence value may cause anxiety if independence is threatened (Schwartz, 2012a). In general, it seems that values and emotions that share the same goals are related (Nelissen et al., 2007). Tamir et al. (2016) have argued that values can also set standards for emotions, not just behaviors, by pointing out desirable emotions. For instance, the more self-enhancement values were endorsed the more respondents wanted to feel pride in their everyday life. In the teaching context, Büssing et al. (2020) found that universalism and benevolence predicted the anticipated enjoyment of teaching. Van Boven et al. (2010) claim that teachers tend to feel different emotions in class maybe because of values. However, studies examining relationships between value priorities and emotions among teachers are scarce.

### Aim and research questions

The aim of this study is to elaborate the location of Turkish teachers’ rational and non-rational truth values in Schwartz’s value structure in correlation with emotions in teaching. For this purpose, the following research questions are presented:

1. What is the location of Turkish teachers’ truth-related values in the Schwartz value structure?
2. Do the value priorities among Turkish teachers differ by gender? If yes, how do they differ?
3. What are the relationships between personal values and emotions among Turkish teachers?

## Methods

### Research context

Teachers work at private and state schools in Türkiye whose compulsory education lasts 12 years (4 years in each of the three stages of primary education, elementary education, and secondary education). All students who have completed their compulsory education must take a national exam to be enrolled in a higher institution. Turkish Higher Education Institute distributes the students according to their scores and choices that they made after the national exam. Student teachers start their academic life when they are enrolled in a faculty of education. They must enter a national exam after their graduation and obtain a satisfied score for their appointment in a state school. The graduated teacher students can work at any private school if accepted. The students who graduated from institutions or other faculties must obtain a teaching license from a faculty of education to become formal teachers.

### Participants

Altogether 293 Turkish teachers participated in the study. After the analyses, 14 of the respondents were discarded because of their deficient and inadequate information. The final sample consisted of 279 Turkish teachers, 64 of whom were male (23%) and 215 were female (77%). The respondents worked in private or state schools (87%) in varied cities located in Türkiye. The mean age of the participating teachers was 36.8 years {with Std = 8.04 [female 35.5 (Std = 8.00) and male 41.1 (Std = 8.58)]}. Their teaching experience varied between 1 and 40 years and the mean years of teaching experience was 13.02 (Std = 8.58). The minority (15%) were classroom teachers, and the majority (85%) were subject teachers. Most of the teachers held bachelor’s degrees (72%).

### Instruments

#### Portrait value questionnaire

To measure value priorities, the Portrait Value Questionnaire (PVQ; Schwartz et al., 2001) was used. PVQ was conducted in Turkish language which was adapted by Demirutku and Sumer (2010). It consisted of 40 items which represented 40 different people, in other words, 40 portraits. In each portrait, a value type was described in two sentences. For example, ‘He thinks it is important to be ambitious. He wants to show how capable he is.’ or ‘It’s very important to her to help the people around her. She wants to care for their well-being.’ The respondents made a judgment on how similar they are to the people in the characterized portraits on a six-point scale (1 = not like me at all; 6 = very much like me). Since the Turkish language has a single word for gender pronouns (she or he), one version of the questionnaire (Turkish version of PVQ) was used for both female and male participants.

In this study, additional (truth-related) items were translated into Turkish by the researcher. Then, two English teachers translated the items and made a co-decision on appropriate translations after back-translation procedures. In this data, Cronbach’s alphas for PVQ were 0.70 for power, 0.83 for achievement, 0.87 for hedonism, 0.72 for stimulation, 0.87 for self-direction, 0.95 for universalism, 0.80 for benevolence, 0.59 for tradition, 0.78 for conformity, 0.84 for security, 0.83 for rational truth and 0.25 for non-rational truth. Centralized sum variables were employed in the study to prevent unequal usage of the scale: a personal mean of all 40 portraits was determined for each subject independently, and the means of the sum variables were divided by the personal mean.

#### Teacher emotions scale

Emotions in teaching were measured using the Teacher Emotions Scale (TES; Frenzel et al., 2016) which was adapted into Turkish by Alpaslan and Ulubey (2017). The TES, a 4-point Likert scale (0 = strongly disagree, 4 = strongly agree), consisted of 12 items as a list of statements describing teachers’ experiences in teaching. In this data, Cronbach’s alphas for TES, which is based on three dimensions, are 0.80 for anger, 0.75 for anxiety, and 0.93 for enjoy. Teacher emotions measure teachers’ emotional experiences in teaching; for example, ‘I generally have so much fun teaching that I gladly prepare and teach my lessons’ or ‘I generally feel tense and nervous while teaching’.

### Procedure

The online questionnaire was delivered to the volunteering teachers via e-mail after obtaining ethical permission from a state university in Türkiye. The data were collected online in summer 2021. Strict confidentiality was followed in the treatment and reporting of the data without any identifier. The raw data is only available to the researchers and protected in encrypted files. Completing the whole questionnaire took approximately 20 min.

### Data analysis

In this cross-sectional correlational study, the data was analyzed using SPSS software package version 26.0. The mean and standard deviation of values show the differences between females and males, MDS scaling introduces the positive and negative correlations between values, and the intercorrelation demonstrates the significance of the differences between values and emotions. The circular structure of the Schwartz Value Theory and how truth-related values are located in it was tested by multidimensional scaling. Due to the violence of the normal distribution of value and emotion variables, the mean differences in values between genders were tested with the non-parametric Mann–Whitney *U* test. The Pearson correlation was used to examine the correlations between values and emotions.

## Results

### The location of truth-related values in the Schwartz value structure

**Figure 2:**
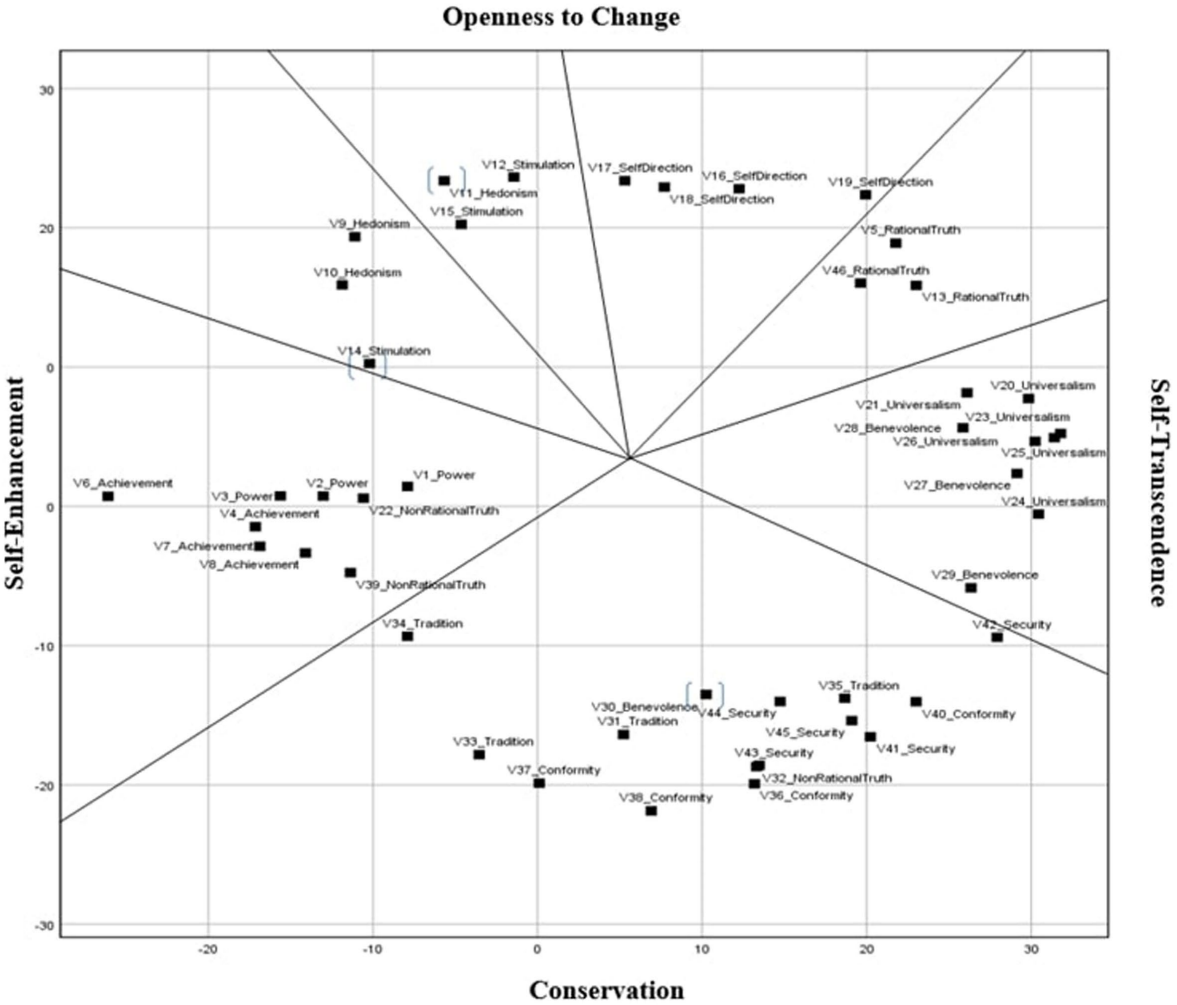
Multidimensional scaling of 46 items (MDS, PREFSCAL). *N* =279. The mislocated items are shown in parentheses.

In order to test whether the circular structure by Schwartz (1992) Value Theory is observed in our sample, SPSS multidimensional scaling (MDS, PREFSCAL) was performed. In MDS schemas, values are represented as points on a multidimensional space, and the distance between points reflects the relationships between values (Kusdil and Kagitcibasi, 2000). In other words, values located in close proximity to each other on a multidimensional space (e.g., benevolence and universalism) are expected to be conceptually positively related and values that are far away from each other (e.g., rational truth and non-rational truth) are expected to show low or negative correlation with each other. In Figure 2, the location of the values shown in the multidimensional space symbolizes their conceptual relationship with other values.

The results broadly confirmed the theoretical model outlined by Schwartz. However, unlike the theoretical model, it was observed that some value types were combined with the value types adjacent to them. Such small deviations are considered quite normal because it was reported that samples in which all 10 value types are detected in distinct locations are very rare (Sagiv and Schwartz, 1995). In this sample, it was found that four values (hedonism/stimulation, self-direction, and rational truth) of the 12 value types (values and added truth-related values) had a distinct location on the multidimensional space, while the rest were combined with a value type on the side. No discernible value was detected in the theoretical sequence, and all values were observed in Schwartz’s value structure. And there was no complication with the differentiation of the main value groups (self-enhancement, openness to change, self-transcendence, and conservation).

There were some deviations from Schwartz’s model, but mostly the basic value types followed the theory. The most glaring deviation in the value circle in this sample was observed in the non-rational value type. Non-rational truth is located in a fairly dispersed way (close to achievement-power and security-conformity), which was expected in light of previous studies (Ahola, 2017). Nevertheless, this confirmed our hypothesis in the Schwartz value model since these values were located far away and on the opposite side of the rational truth value type.

The fact that the two additional values were located at opposite poles indicates that they conflict with each other as hypothesized. Rational truth value was found to be congruent to the self-direction and self-transcendence types as predicted in the theory. The scattered inclusion of the non-rational items in the theoretical sequence might be a situation specific to this value type or the result of a situation specific to Turkish culture.

### Gender differences in values

Table 2 reports the rank of the values and differences in value priorities according to gender. Universalism and rational truth were highest in rank. Power and non-rational values were the least value preferences by teachers. However, those outside of self-direction and hedonism were not significant in gender. And enjoyment had the highest mean among emotions in teaching. It was observed that there were slightly higher scores in females’ emotions by comparison with males.

**Table 2:** Means (M) and standard deviations (SD) for gender.

Values and Emotions	Female (*N* = 215)		Male (*N* = 64)		
	*M*	SD	*M*	SD	*p*
Universalism	1.18	0.19	1.20	0.17	ns
Rational truth	1.13	0.17	1.12	0.16	ns
Benevolence	1.07	0.16	1.06	0.14	ns
Self-direction	1.11	0.15	1.04	0.17	0.00**
Security	1.06	0.16	1.07	0.12	ns
Conformity	1.02	0.16	1.04	0.16	ns
Hedonism	1.01	0.26	0.95	0.29	0.05*
Stimulation	0.95	0.23	0.90	0.20	ns
Tradition	0.87	0.20	0.92	0.21	ns
Achievement	0.86	0.29	0.90	0.27	ns
Non-rational truth	0.80	0.24	0.75	0.22	ns
Power	0.68	0.35	0.73	0.31	ns
Enjoyment	3.39	0.51	3.32	0.49	ns
Anger	1.82	0.51	1.74	0.46	ns
Anxiety	1.70	0.53	1.61	0.48	ns

*N = 279.*

ns, not significant, *p < 0.05, **p < 0.01.

Mann–Whitney *U* test results (Table 2) showed that hedonism (*p* = 0.05) and self-direction (*p* = 0.002) values were significant in genders. The results were interpreted that females attributed importance to hedonism [mean = 1.01 (female); mean = 0.95 (male)] and self-direction [mean = 1.11 (female); mean = 1.04 (male)] more than males. However, the results showed that there was no significant difference between females and males regarding emotions.

### Relationships between values and emotions

The correlations between values and emotions were reported in Table 3. There we can see that enjoyment was negatively correlated with anger and anxiety which had a high-sized positive correlation between each other. Enjoyment also had a negative correlation with non-rational truth. Anger showed negative correlation with conformity, security, and benevolence, and positive with power and achievement. Anxiety and security had an inverse relation while anxiety and non-rational truth and tradition were positively correlated.

**Table 3:** Inter-correlation among values and emotions.

	1	2	3	4	5	6	7	8	9	10	11	12	13	14
1. Power														
2. Achievement	0.40**													
3. Tradition	−0.16**	−0.17**												
4. Conformity	−0.28**	−0.14*	0.25**											
5. Security	−0.35**	−0.22**	−0.01	0.42**										
6. Universalism	−0.34**	−0.45**	−0.14*	0.01	0.16**									
7. Benevolence	−0.39**	−0.40**	−0.00	0.12*	0.07	0.39**								
8. Self-direction	−0.25**	−0.31**	−0.33**	−0.22**	−0.01	0.25**	0.15**							
9. Stimulation	0.00	−0.04	−0.22**	−0.44**	−0.39**	−0.23**	−0.17**	0.24**						
10. Hedonism	0.10	−0.02	−0.26**	−0.35**	−0.26**	−0.26**	−0.29**	0.03	0.40**					
11. Rational Truth	−0.30**	−0.29**	−0.35**	−0.05	0.21**	0.48**	0.18**	0.35**	−0.08	−0.04				
12. Non-rational Truth	0.07	0.00	0.27**	−0.20**	−0.31**	−0.32**	−0.16**	−0.15*	0.10	0.00	−0.36**			
13. Enjoyment	−0.10	−0.07	−0.06	0.08	0.10	−0.03	0.09	0.09	0.09	−0.01	0.11	−0.22**		
14. Anger	0.17**	0.13*	0.00	−0.17**	−0.14*	−0.09	−0.18**	0.00	0.04	0.04	−0.05	0.10	−0.52**	
15. Anxiety	0.06	0.03	0.14*	−0.11	−0.16**	−0.00	−0.08	−0.05	0.06	−0.05	−0.08	0.20**	−0.47**	0.56**

*p < 0.05, **p < 0.01.

## Discussion

### Results in the light of the previous literature

This research aimed to reveal the location of Turkish teachers’ rational and non-rational truth values in Schwartz’s value structure in correlation with emotions in teaching. Three research questions demonstrate; (1) the MDS results of rational and non-rational truths in the circular continuum, not surprisingly, located reversed among the consistent ordering of 10 sets of values, (2) Turkish female and male teachers have some distinct traits in hedonism and self-direction which does not represent the original theory in gender, and (3) the empirical evidence of teaching emotions which supports the predictive and explanatory dynamics of values in relation to emotions.

Firstly, it is legitimate in this research that non-rational truth is scattered in the structure since past research also indicated it was not located in a distinct region but opposite to rational truth (Ahola, 2017). However, rational truth emerged between self-direction and self-transcendence as a distinct region. The unique location of rational truth and its reverse location of non-rational truth revealed that PVQ perfectly reproduced the order of teachers’ values around the circle of 10 values in the original theory. Since education values show much similarity with rational truth (Portman, 2014), the current MDS result supporting fine-tuned partitioning provides a precise understanding of the relations between values and education. This implies rational truth presents some motivation in common with self-direction, universalism, and benevolence in education. Our research result also confirms the previous research (Kusdil and Kagitcibasi, 2000) in which Turkish teachers’ universalism and self-direction were positively correlated with secularism whose meaning embraces rational thought (Tejani, 2013).

Secondly, the fact that the value of hedonism, which is a value for enjoying life and was significantly higher in female teachers, may indicate that female teachers try to see the good sides of life more than male teachers. Likewise, the fact that women teachers had higher self-direction values may indicate that they think more individually and that they care more about the responsibility of standing on their own feet in the teaching profession. When Schwartz’s value theory is evaluated here, the value confusion of male and female teachers in Türkiye draws attention since our result does not support the previous studies with teachers (Karabacak et al., 2019; Ros et al., 1999; Tekin, 2021). According to Boratav (2009), why hedonism is highly valued by Turkish females is because gaining pleasure and enjoying life are considered feminine behaviors within Turkish culture. The same contradiction was observed in some previous studies with teachers (Firat, 2008; Memis and Gedik, 2010; Turkan and Kaya, 2019) and described as a reflection of Turkish culture on women teachers who are influenced by both the Western and the Eastern culture due to the geopolitical location of Türkiye. Since females are described as having the social role of “being a mother” or “being a good wife” (Firat and Acikgöz, 2012), teaching occupation is generally regarded as a traditional female occupation in Türkiye (Gonluacik et al., 2022). Thus, a possible explanation for hedonism and self-direction might be that Turkish female teachers tend to be stripped of the expected gender roles of today’s society and enjoy life independently. The increased education level in society results in females becoming more liberated which may result in females valuing hedonism highly more than males (Dirilen-Gumus and Buyuksahin-Sunal, 2012; Feather, 2004). Dirilen-Gumus and Buyuksahin-Sunal (2012) also argue individuals’ tendency to self-direction indicates their tendency to get more individuated; therefore, our results may indicate that female teachers are getting more individuated. Hedonism and self-direction inherently may become more important to female teachers since the importance of certain values increases for one sex as changing societal conditions facilitated their expression and pursuit (Schwartz and Rubel-Lifschitz, 2009).

Finally, correlation differences between values and emotions confirm one of the main features of values defined in Schwartz’s theory that values are inextricably linked with emotions (Schwartz, 2006). We discovered that teachers’ enjoyment in teaching was negatively correlated with non-rational truth. Not surprisingly as evidenced in previous studies (Malouff et al., 1992; Thyer et al., 1987) anxiety was positively correlated with non-rational truth. These results make sense conceptually, considering that feelings of enjoyment and anxiety are strongly negatively related to each other. Feelings of enjoyment refer to pleasure and satisfaction with something that is achieved (Frenzel, 2014) and anxiety refers to a perception of a threat to ego or self-esteem and not being able to cope with it (Smith and Lazarus, 1993). Non-rational truth values represent a belief in intuitiveness and denial of rationality (Ahola, 2017). Thus, non-rational truth may prompt worry about the unpredictability of the world, compared to the rational truth that emphasizes theoretical, logical, and predictable truth (Wach and Hammer, 2003). This result captures a meaningful understanding of values that non-rational truth may reduce teachers’ enjoyment and even cause anxiety in teaching.

In conclusion, the current study demonstrated the location of the truth-related values in Schwartz’s value structure. The findings support Ahola’s (2017) research results in which rational truth values were positively associated with self-direction and self-transcendence. The teachers prioritizing rational truth concern that material well-being is secondary or unnecessary for the welfare or exploring. And as demonstrated in the previous study (Ahola, 2017), non-rational truth values were positively correlated with power and achievement, which indicates that those teachers attach importance to being strong or successful in the hierarchy within the group they are affiliated with. This result could be due to the Turkish culture, where social orderliness is predominantly achieved through hierarchical roles as Kusdil and Kagitcibasi (2000) inferred. On the other hand, correlations revealed the positive relation between anxiety and negative relation between enjoyment and non-rational truth. After all, teachers’ values could promote their emotions in teaching (Hagenauer and Volet, 2014; Schutz, 2014). Hence, our study provides some implications that teacher education programs may improve teachers’ understanding of quality teaching skills and knowledge by value-based pedagogy (Curtis, 2012). It is essential that teacher education programs systematically enhance identifying and clarifying one’s own values as a basis for the profession and underlying professional actions. This has been shown to be essential, but a bit neglected aspect in teacher education. The effectiveness of teacher preparedness could be further promoted considering the relationship between teachers’ non-rational truth and emotions in teaching as evidence in this study. It is also important to emphasize the influence of teachers’ well-being which could benefit from teachers’ values (Barni et al., 2019) and their emotions in teaching (Frenzel et al., 2015) in requirement of teacher quality.

### Methodological reflections and limitations

The current study has investigated the location of truth-related values in relation to emotions in a sample of Turkish teachers. Despite the contribution to the literature on teachers’ values and emotions, the limitations of this study are in question and future researchers should always pay regard to them in interpreting the presented results. The data were collected at one time and shortly after the lockdown. When we consider the stability of values across time distinguishes them from needs and motives and therefore change more readily (Sagiv and Schwartz, 2022), both values and emotions could have been influenced during the lockdown. Possible factors that would affect teachers’ emotions or the relationship between them in a teaching process that they had no previous experience with were ignored. However, since the current research was applied to the teachers in Türkiye which constitutes a tight culture (Gelfand et al., 2011), it will be worthwhile for future work to include diverse societies to compare teachers’ values and emotions in teaching in tight and loose nations. The current study, which is original in its field, will undoubtedly shed light on future studies. Future research of this article generates an interesting study on the location of teachers’ truth-related values in Schwartz’s value structure and the relationship of them with emotions from the point of gender differences. Since teachers’ emotions are key components of teachers’ psychological well-being (Frenzel et al., 2015), teacher training programs might benefit the positive relationship between rational truth values and enjoyment in teaching. Moreover, because emotions are essential for teachers’ work and outcomes of teaching, in teacher education it is essential to help pre-service teachers to find adaptive ways to cope with negative emotions such as anger and anxiety. Since teachers’ value priorities can vary with culture (Fischer and Schwartz, 2011), in-depth cross-cultural studies may also be conducted in tight and loose cultures exploring gender-based similarities and differences to enhance the generalizability of the findings of the study.

On the other hand, the fact that item 42, which is related to national security, was located near the value dimension of self-transcendence demonstrates the teachers associate national security with benevolence and universalism. Item 11 and item 14, which are about enjoying life and enjoying adventure, were dislocated between hedonism and stimulation. This finding is plausible when we examine their meaningful content. And item 30, caring about forgiveness, was located close to conformity and tradition, rather than within benevolence, which indicates that the teachers might perceive forgiveness as a factor of adapting to the environment in a communitarian culture as Türkiye (Sargut, 2015).

Lastly, even if we did not find any meaningful difference between value priorities and age, there exists a meaningful age difference in hedonism between males and females without using centralized sum variables. However, it does not affect our result as it was systematical in research that hedonism was negatively associated with age without a moderation effect of culture (Borg et al., 2017; Robinson, 2013), younger teachers also had higher regard for hedonism than older ones in our study. And the reliability of some of the values was low due to the tiny number of entries in each value’s index. However, significantly lower reliabilities have also been seen in other studies (Myyry and Helkama, 2001; Sagiv and Schwartz, 1995). Nevertheless, the correlations between the values and other variables confirm the sinusoid curve hypothesis (Schwartz, 1992). Thus, despite the weaknesses in the Cronbach alphas, the pattern of correlations suggests the reliability of the measure.

## Conclusion

As a result of this study, the location of rational and non-rational truth values in the value structure confirms the previous results (Ahola, 2017). It was proved that rational and non-rational values had a confirmed location among global values. Considering that teachers are important socialization agents in their cultures (Barni et al., 2018; Schwartz, 1992; Tamm et al., 2020) and role models to children (Thornberg and Oguz, 2016), the high regard of the rational truth value – being the second highest in the value hierarchy for both females and males – and its positive link to self-transcendence values universalism and benevolence, are notable. Self-transcendence values aim to promote the welfare of others (Schwartz, 1992), and especially universalism is related to mature moral thinking (Myyry, 2022). Rational truth emphasizes theoretical, logical, and predictable truth instead of intuitive thinking characterized by non-rational truth (Wach and Hammer, 2003). Thus, the results suggest that teachers at least in our sample emphasize transmitting collective instead of personal interests (Schwartz, 1992) and the importance of rational thinking. Nevertheless, how endorsing rational or non-rational truth values is associated with professional practices of teachers is an interesting question that should be scrutinized in future research.

## Data Availability

The raw data supporting the conclusions of this article will be made available by the authors, without undue reservation.

## Footnotes

^1^Türkiye is the official international name for the country formerly known as Turkey.
